# Draft Genome Sequence of Acidianus ambivalens DSM 3772, an Aerobic Thermoacidophilic Sulfur Disproportionator

**DOI:** 10.1128/MRA.01415-19

**Published:** 2020-01-16

**Authors:** Emma Bertran, Lewis M. Ward, David T. Johnston

**Affiliations:** a Department of Earth and Planetary Sciences, Harvard University, Cambridge, Massachusetts, USA; b Department of Geosciences, Princeton University, Princeton, New Jersey, USA; c Earth-Life Science Institute, Tokyo Institute of Technology, Tokyo, Japan; University of Southern California

## Abstract

Here, we describe the genome sequence of Acidianus ambivalens DSM 3772, an archaeon belonging to the Sulfolobales order that was first isolated from continental solfataric fields. This thermoacidophile was sequenced because it utilizes a unique sulfur disproportionation pathway that enables this metabolism under aerobic conditions, in contrast to obligately anaerobic bacterial sulfur disproportionators.

## ANNOUNCEMENT

Acidianus ambivalens DSM 3772 is an obligate chemolithoautotrophic acidophile isolated from continental solfataric fields (1, 2). It belongs to the *Sulfolobales* order within the archaeal domain and grows most optimally at 80°C and a pH of 1 to 3 (1, 2). This archaeon has been suggested to cope with low pH levels by an extreme turnover of its terminal oxidase, which in turn generates a proton gradient by chemical charge separation (3). Under aerobic conditions, this archaeon performs oxygen-dependent elemental sulfur disproportionation to sulfide and sulfite (4). Under anaerobic conditions, *A. ambivalens* uses hydrogen as an electron donor for elemental sulfur reduction (5). It has also been shown to grow anaerobically with tetrathionate as the sole sulfur substrate (5).

Genomic DNA of *A. ambivalens* was received from the DSMZ following growth in medium 358a and extraction via a JetFlex genomic DNA purification kit from Genomed. DNA libraries were prepared using a Nextera XT library prep kit on a Hamilton Microlab Star automated liquid-handling system prior to sequencing via the Illumina HiSeq platform using a 250-bp paired-end protocol. Reads were adapter trimmed with Trimmomatic v0.30 (6). *De novo* assembly was performed using SPAdes v3.7 (7), and annotation was performed using RAST v2.0 (8). The publicly available genome was annotated with PGAP (9). CheckM v1.0.12 (10) was used to estimate genome completeness. MetaPOAP v1.0 (11) was used to determine the likelihood for the presence or absence of metabolic pathways. Taxonomic assignment of the genome was verified with GTDB-Tk v0.3.2 (12). Hydrogenase proteins were classified with HydDB (13). Default parameters were used for all software.

The *A. ambivalens* genome was recovered at 140× average coverage as 721,210 reads, which were assembled into 65 contigs with an *N*_50_ value of 1,228,068 bp and totaling 2,326,940 bp encoding 2,794 coding sequences and 48 RNAs. The genome has a 34.4% GC content. CheckM estimates the genome to be 100% complete based on the presence of single-copy marker genes with 0% redundancy and strain heterogeneity.

The aerobic sulfur disproportionation pathway encoded by *A. ambivalens* differs from that of anaerobic sulfur disproportionators, which encode a variant of the dissimilatory sulfate reduction pathway (5, 14, 15). Aerobic elemental sulfur disproportionation to sulfite and sulfide in *A. ambivalens* is promoted by a cytoplasmic sulfur oxygenase reductase (SOR), with neutral or slightly acidic pH optima (5 to 7.4) (16). The genes encoding thiosulfate quinone oxidoreductase (TQO) (subunits *doxA*, *doxB*, *doxC*, *doxD*, *doxE*, and *doxF*), argued to be involved in the formation of tetrathionate from thiosulfate, a side product of the nonenzymatic condensation of HSO_3_^2−^ with S^0^ (16), were also found, consistent with previous reports (3).

Genes encoding the two subunits of tetrathionate hydrogenase (*tth1* and *tth2*) were found in the genome. Tetrathionate hydrogenase is soluble, extracellular, and acidophilic, and it is essential for growth with tetrathionate via its disproportionation to sulfate, thiosulfate, and sulfite (5).

Genes encoding sulfur/polysulfide reductase (*sreA*, *sreB*, *sreC*, *sreD*, and *sreE*) were also found. *A. ambivalens* also carries genes encoding a number of NiFe hydrogenases (*hydS*, *isp1*, *isp2*, *hydL*, *hypD*, *hypC*, and *hoxM*), which hold a central role in electron-donating reactions, including sulfur/polysulfide reduction. These hydrogenase enzymes have been shown to exhibit a high optimum pH, which is puzzling given the low-pH environments in which this archaeon optimally grows (17).

### Data availability.

This whole-genome shotgun project has been deposited at DDBJ/ENA/GenBank under the accession number WHYS00000000. The fastq files of the raw reads were deposited in the NCBI SRA under accession number SRR10430294.
